# *EZH2*-mediated H3K27me3 is a predictive biomarker and therapeutic target in uveal melanoma

**DOI:** 10.3389/fgene.2022.1013475

**Published:** 2022-10-06

**Authors:** Chen Hou, Lirong Xiao, Xiang Ren, Lin Cheng, Bo Guo, Meixia Zhang, Naihong Yan

**Affiliations:** ^1^ Research Laboratory of Ophthalmology, West China Hospital, Sichuan University, Chengdu, China; ^2^ Department of Ophthalmology, West China Hospital, Sichuan University, Chengdu, China; ^3^ Department of Ophthalmology and Visual Sciences, University of Iowa, Iowa City, IA, United States

**Keywords:** EZH2, H3K27me3, uveal melanoma, UNC1999, ferroptosis

## Abstract

Although gene mutations and aberrant chromosomes are associated with the pathogenesis and prognosis of uveal melanoma (UM), potential therapeutic targets still need to be explored. We aim to determine the predictive value and potential therapeutic target of *EZH2* in uveal melanoma. Eighty-five uveal melanoma samples were recruited in our study, including 19 metastatic and 66 nonmetastatic samples. qRT-PCR, immunohistochemistry staining, and western blotting were applied to detect the expression of EZH2 and H3K27me3*.* We found that EZH2 (41/85, 48.24%) and H3K27me3 (49/85, 57.65%) were overexpressed in uveal melanoma. The expression of EZH2 was not significantly associated with metastasis. High H3K27me3 expression was correlated with poor patient prognosis. UNC 1999, an EZH2 inhibitor, can downregulate H3K27me3 expression and has the most potency to inhibit OMM1 cell growth by the cell cycle and ferroptosis pathway. These results indicate that H3K27me3 can be a biomarker predicting a poor prognosis of UM. *EZH2* is the potential therapeutic target for UM.

## Introduction

Uveal melanoma (UM), originating from melanocytes, is one of the most common melanomas after cutaneous melanoma. Metastases occur in approximately 50% of UM patients, and about 90% of metastatic foci involve the liver with a poor prognosis (Adriana et al., 2017). Once metastasis occurs, the survival time will not exceed one year (Yonekawa and Kim, 2012). In the past 30 years, although primary UM was treated positively, the 5-year survival rate of UM has not improved (Carvajal et al., 2017).

Enhancer of zeste homolog 2 (*EZH2*) is the functional enzymatic component of the polycomb repressive complex 2 (PRC2), which collaborates with polycomb repressive complex 1 (PRC1) to inactivate gene expression (Kahn et al., 2016). *EZH2* catalyzes the trimethylation of histone 3 lysine 27 (H3K27me3), leading to the repression of gene transcription and regulating proliferation and differentiation in the growth process (Barsotti et al., 2015).

*EZH2* is highly expressed in various tumors. The overexpression of *EZH2* is related to worse survival in NK/T-cell lymphoma, prostate cancer, breast cancer, cutaneous melanoma, gastric cancer, endometrial cancer, and esophageal squamous cell carcinoma (Bachmann et al., 2006; Wei et al., 2008; Cai et al., 2010; Liu et al., 2019). Even though *EZH2* overexpression and gain-of-function present oncogenic activity, *EZH2* also plays a crucial role in tumor suppression, such as myelodysplastic syndromes, myeloproliferative neoplasms, and T cell acute lymphoblastic leukemia (Ernst et al., 2010; Nikoloski et al., 2010; Ntziachristos et al., 2012). Therefore, it is necessary to identify the overexpression of *EZH2* and its therapeutic potential in uveal melanoma.

In addition to trimethylation, PRC2 also mediates mono and dimethylation of H3K27 (Peters et al., 2003). In the progress of EZH2 methylating H3K27, it can cause increased H3K27me3 with a subsequent decrease in H3K27me1 and H3K27me2 (McCabe et al., 2012; Barsotti et al., 2015). Whereas more than 50% of total histone H3 is H3K27me2 and H3K27me3, monomethylated H3K27 (H3K27me1) are substantially less abundant at about 10%–20% (Jung et al., 2010). The widespread deposition of H3K27me2 is believed to protect target loci from histone acetyltransferases (HATs) activity, ensuring proper control of cell type-specific enhancement (Jung et al., 2010). H3K27me3 is an epigenetic hallmark of gene silencing but not H3K27me1 and H3K27me2 (Liu and Liu, 2022).

However, the indications of the level of H3K27me3 also have two sides in different cancers. Indeed, H3K27me3 is expressed at low-level in breast, ovarian, pancreatic cancers, meningiomas, and malignant peripheral nerve sheath tumors, and a reduction in H3K27me3 is a predictor of the poor prognosis in patients with carcinoma (Wei et al., 2008; Schaefer et al., 2016; Katz et al., 2018). But H3K27me3 is elevated in hepatocellular carcinoma, nasopharyngeal carcinoma, endometriosis, and prostate cancer, predicting the poor prognosis (Cai et al., 2011a; Cai et al., 2011b; Colón-Caraballo et al., 2015; Liu et al., 2016; Ngollo et al., 2017).

Although many studies have described *EZH2* and H3K27me3 in different cancers, it is unclear whether the *EZH2* mutation exists in uveal melanoma and the effect of *EZH2* and H3K27me3 in uveal melanoma. There have been two recent studies on EZH2 expression in UM. Cheng and colleagues examined the expression level of EZH2 in 89 samples by IHC, suggesting that high EZH2 expression was significantly associated with metastasis (Cheng et al., 2017). Jin et al. (2020) found that overexpression of EZH2 promoted the growth, migration, and invasion of UM. Our study investigated the association between EZH2 and H3K27me3 expression and prognosis. With the development of selective EZH2 inhibitors, we attempt to reveal the roles of EZH2 and H3K27me3 in uveal melanoma *via* small-molecule EZH2 inhibitors, including GSK126, GSK503, EED226, UNC1999, and EZP6438.

## Materials and methods

### Sample selection

The inclusion criteria was as follows: 1) Uveal melanoma patients who underwent global enucleation in the Department of Ophthalmology in China West Hospital from 2009 to 2017; 2) We can follow up on the endpoint (metastasis or non-metastasis) by telephone. Eventually, 85 tumor samples from uveal melanoma patients, all formalin-fixed and paraffin-embedded (FFPE), 19 metastatic and 66 nonmetastatic, were obtained from the Department of Pathology in China West Hospital. Patient demographics, metastasis, and clinical and pathological data were obtained from the clinical records. For our study, the presence of metastasis was regarded as the endpoint according to telephone follow-up. All participants gave informed consent before the examination. The Ethics Committee of West China Hospital, Sichuan University, approved this study.

### Immunohistochemical staining and evaluation

Immunohistochemistry was performed on 5-μm-thick formalin-fixed, paraffin-embedded (FFPE) tissue sections. Sections were put in 0.2% potassium permanganate solution for 20 min and 1% oxalic acid solution in 10 s for depigmentation, following deparaffinization and antigen retrieval. Sections were incubated at 4°C overnight with EZH2 Rabbit Antibody (1:1000, Cell Signaling Technology, 3147, Beverly, MA, United States) and H3K27me3 Rabbit Antibody (1:1000, Cell Signaling Technology, Beverly, 9733, MA, United States). We used Vulcan Fast Red Chromogen Kit 2(Biocare Medical, 1-800-799-9499) as a chromogenic reagent. The slides were scanned automatically by a NanoZoomer Digital Pathology System. The acquired pictures were viewed with NDP view software. We counted 1,000 cells and recorded the percentage of positive cells. We discretized the continuous data of EZH2-positive cell percentage to the categorical data from 0 to 3 (0, no staining; 1, ≤10%; 2, ≤30%; 3, >50%). The results were then subdivided into low (score = 0, 1) and high (score = 2, 3) expression groups.

### Cell culture

The UM OMM1 cells were kindly provided by Dr. M. J. Jager, Leiden University Medical Center, Leiden, and Dr. Renbing Jia, Department of Ophthalmology, Shanghai Ninth People’s Hospital, Shanghai Jiaotong University School of Medicine. ARPE cell line was gifted by Dr. Kang Zhang, West China Hospital, Sichuan University. Cultured explants were either exposed to GSK126 (Selleckchem, S7061), GSK503 (Selleckchem, S7804), EED226 (Selleckchem, S8496), UNC1999 (Sigma Aldrich Corp, SML0778), and EPZ6438 (Selleckchem, S7128).

### Western blot

Primary antibodies included β-actin (1:2000; Abcam, ab6276), histone H3 (1:1000, Cell Signaling Technology, 4499), EZH2 (1:1000, Cell Signaling Technology, 3147), H3K27me3 (1:1000, Cell Signaling Technology), and H3K27ac (1:1000, Cell Signaling Technology). The secondary antibodies were LI-COR fluorescent antibodies (1:10000, LI-COR 926–32210, LI-COR 926–32211, LI-COR 926–68072). The images were captured on the Odyssey CLx Infrared Imaging System (LI-COR Biosciences, Lincoln, NE, United States). Western blotting bands were quantified using Odyssey CLx v2.1 software.

### Quantitative real-time PCR

All samples were tested in duplicate to examine the mRNA expression, and the average Ct values were used for quantification. The primer sequences are shown in Supplementary Table S1.

### Iron staining

Cells were treated with or without 10 μM UNC1999 for 48 h in glass bottom dishes. Then the cells were washed three times with HBSS followed by incubation of 1 μM Ferro orange (Dojindo, Japan) in HBSS for 30 min at 37°C and 5% CO_2_. And then observe and photograph immediately using Super Resolution Microscope (N-SIM S, Nikon).

### Measurement of cellular reactive oxygen species

The level of reactive oxygen species was measured using a Reactive Oxygen Species Assay Kit (Beyotime Biotechnology, China). Cells were seeded in 96-well plates and treated with 10 μM UNC1999 for 48 h. The cells were incubated with 10 μM DCFH-DA for 20 min at 37°C and then measured at 488 nm excitation and 525 nm emission by a fluorescence spectrophotometer (BioTek).

### The measure of malondialdehyde and glutathione

The malondialdehyde (MDA) level was measured using MDA Assay Kit (Dojindo, Japan) per the manufacturer’s instructions. The glutathione (GSH) level was measured using GSSG/GSH Quantification Kit II(Dojindo, Japan), following the manufacturer’s instructions.

### Transmission electron microscopy

Cells were collected in a centrifuge tube after treatment with 10 μM UNC1999 for 48 h and immediately fixed in 0.5% phosphate-glutaraldehyde. Lilai biomedicine experiment center post-fixed, embedded, cut, and imaged.

### JC-1

Cells were treated with or without 10 μM UNC1999 for 48 h in glass bottom dishes. The JC-1 Mitochondrial Membrane Potential Assay Kit (MCE, China) was used to detect mitochondrial membrane potential.

### Mitochondrial respiration mito stress test

Mitochondrial respiration mito stress tests were performed using XFe24 Seahorse Mitochondrial Respiration Mito Stress Test (Agilent technologies, United States). The assay procedure was performed according to the guidelines from the XFe24 Seahorse Mitochondrial Respiration Mito Stress Test (Agilent Technologies) (Divakaruni et al., 2014).

### Bioinformatic analysis

In RNA-seq analysis, the following parameter settings were applied: *p*-value cut-off = 0.01, log2FC (Fold change) cut-off = 1. Gene ontology (GO) analysis and Kyoto Encyclopedia of Genes and Genomes (KEGG) pathway enrichment analysis were performed in the Database for Annotation, Visualization and Integrated Discovery (DAVID), which is an online bioinformatics tool. Protein-protein interaction (PPI) network analysis was performed based on an online tool, Search Tool for the Retrieval of Interacting Genes (STRING).

### Statistical analysis

Statistical analysis was performed using SPSS, version 23.0. T-test was used when comparing the means of precisely two groups. ANOVA and Chi-Squared tests were used when comparing the means of more than two groups. The correlation between two variables was tested with Spearman’s rho correlation coefficients. We estimated metastasis-free survival by the Kaplan-Meier method. Differences were considered statistically significant when the two-sided *p* value was less than 0.05. Data are expressed as mean ± SD from at least three independent experiments with triple replicates per experiment.

## Results

### Enhancer of zeste homolog 2 and H3K27me3 were overexpressed in uveal melanoma

**FIGURE 1:**
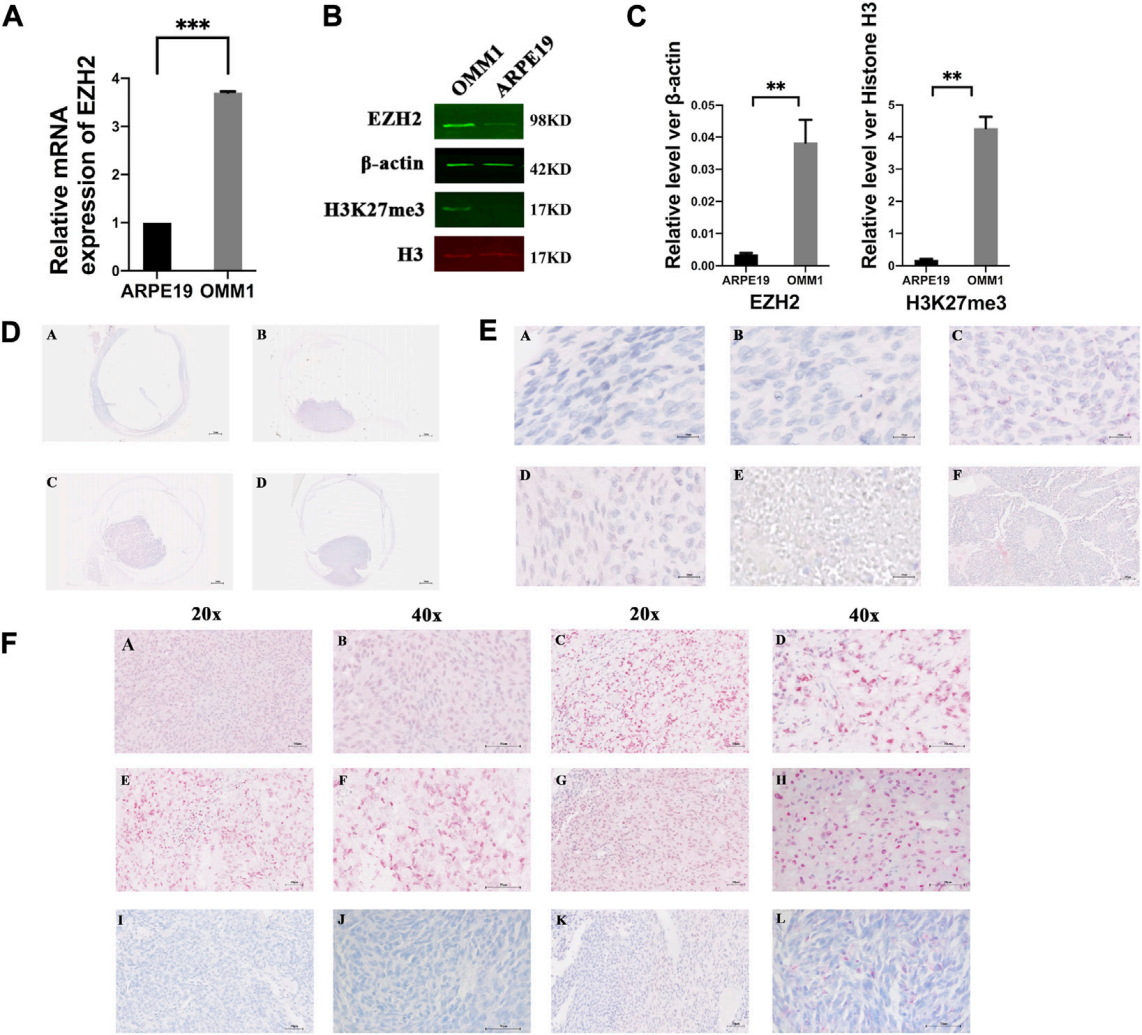
EZH2 and H3K27me3 were overexpressed in uveal melanoma. **(A)** Levels of EZH2 mRNA in OMM1 cells and ARPE19 cells. **(B,C)** Western blot analysis verified overexpression of EZH2 and H3K27me3 in OMM1 cells. The position and background of Histone H3 (680 nm, red) and H3K27me3 (800 nm, green) are the same, and the fluorescence intensity represents the expression of the protein. **(D)** Four kinds of shapes in uveal melanoma. (a) flat-shaped (b) half-dome-shaped (c) dome-shaped (d) mushroom-shaped. **(E)** Five kinds of cell types. (a) spindle A cell type (b) spindle B cell type (c) epithelioid cell type (d) mixed cell type (e) necrosis type (f) Homer-Wright rosettes **(F)** EZH2 and H3K27me3 expression by immunohistochemical staining (a–d) high EZH2 expression; (e–h) high H3K27me3 expression; (i,j) low EZH2 expression; (k,l) low H3K27me3 expression.

To confirm EZH2 expression in UM, we first examined the mRNA expression in ARPE19 and OMM1 cells. The results showed an increased level of *EZH2* mRNA in OMM1 cells (Figure 1A). Western blot analysis also verified overexpression of EZH2 and H3K27me3 in OMM1 cells (Figures 1B,C).

We found different shapes of tumors in uveal melanoma. Figure 1D shows uveal melanomas that are presented as flat- (A), half-dome- (B), dome- (C), and mushroom-shaped (D). The Bruch membranes were ruptured or vanished in half-dome- (B), dome- (C), and mushroom-shaped (D) samples. Unfortunately, due to the unstable paraffin section, it was challenging to observe the tumor’s shape in some sections, so we did not conduct a statistical analysis of the tumor shape.

We showed the spindle A cell type (Figure 1Ea), spindle B cell type (Figure 1Eb), epithelioid cell type (Figure 1Ec), mixed cell type (Figure 1Ed), and necrosis type (Figure 1Ee). Interestingly, Homer-Wright rosettes were detected in one sample, indicating that uveal melanoma cells formed small round shapes around the blood vessels (Figure 1Ef) (Miller et al., 2012; Hakozaki et al., 2013).

**FIGURE 2:**
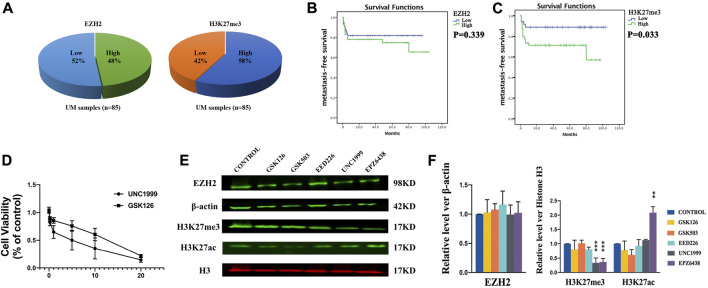
H3K27me3 was associated with prognosis. **(A)** The high expression of EZH2 accounted for 48%, and the high expression of H3K27me3 accounted for 57% in uveal melanoma samples. **(B)** Kaplan-Meier curves showed that expression of EZH2 was not statistically significant with metastasis. **(C)** Kaplan-Meier curves showed that the prognosis of patients with high H3K27me3 expression was worse than that of other patients. **(D)** The growth curves and IC50 values. **(E–F)**. EZH2, H3K27me3, and H3K27ac protein level after treatments of inhibitors of *EZH2* by Western blotting. Among these inhibitors, UNC1999 and EPZ6438 reduce the expression level of H3K27me3. The expression level of H3K27ac was increased after treatment with EPZ6438. The position and background of Histone H3 (680 nm, red) and H3K27me3 (800 nm, green) are the same, and the fluorescence intensity represents the expression of the protein. Data are expressed as mean ± SD from at least three independent experiments with triple replicates per experiment. (*p < 0.05, **p < 0.01).

In the TCGA database, we found the RNA expression level of *EZH2* was correlated with the poor prognosis (Supplementary Figure S1) (Li et al., 2021). In addition, we determined EZH2 and H3K27me3 expression in UM *via* immunohistochemical staining. Figures 1Fa–d shows representative high EZH2 expression in UM samples, and Figures 1Fi,j shows low EZH2 expression. The expression of EZH2 was increased in 41 of 85 (48.24%) UM samples (Figure 2A). In addition, the difference between the high and low expression of EZH2 was not statistically significant with metastasis (Figure 2B).

H3K27me3 was highly expressed in 49 of 85 (57.65%) UM samples (Figure 2A). Figures 1Fe–h shows representative high H3K27me3 expression. Figures 1Fk,l shows low H3K27me3 expression in UM samples by IHC. To confirm the association between H3K27me3 expression and patient survival, we applied statistical analysis and found that H3K27me3 expression was significantly associated with metastasis (*p* = 0.033). Kaplan-Meier curves showed that the prognosis of patients with high H3K27me3 expression was worse than that of other patients (*p* = 0.03) (Figure 2C).

Our study demonstrated that EZH2 and H3K27me3 were overexpressed in uveal melanoma. To determine the correlation of the expression of EZH2 and H3K27me3 in uveal melanoma, we applied Spearman analysis, which demonstrated the expression of EZH2 and H3K27me3 was significantly positively correlated (r = 0.446, *p* < 0.01). The patients with high expression of EZH2 were likelier to have a high expression of H3K27me3.

We applied statistical analysis to determine the association between clinical characteristics and EZH2 and H3K27me3 protein expression. The expression level of EZH2 was higher in those patients older than 50 (*p* = 0.027, OR = 3.531 95% CI 1.155–10.793) (Supplementary Table S2). The clinical characteristics and the expression of H3K27me3 were not statistically significant (Supplementary Table S3).

### Inhibitors of *EZH2* suppress uveal melanoma cell growth

To investigate the importance of *EZH2* in uveal melanoma cells, we examined several small-molecule methyltransferase inhibitors, including UNC 1999, EPZ6438, GSK126, and GSK503, and the embryonic ectoderm development protein (EED) inhibitor EED226 in OMM1 cells. UNC1999 and GSK126 were able to suppress the growth of OMM1 cells effectively. The growth curves and IC_50_ values are shown in Figure 2D. Considering a lower concentration of UNC1999 can effectively inhibit OMM1 cells, we did further investigation. To understand how UNC1999 affected cells, we applied Western blotting to examine the expression levels of EZH2 and H3K27me3*.* Besides, we found that UNC1999 could not reduce the expression level of EZH2 protein but decreased the expression level of H3K27me3 (Figures 2E,F). In comparison, EPZ6438 also reduced the expression level of H3K27me3. However, it could not inhibit OMM1 cell growth. To detect this distinction, we also examined the expression levels of H3K27ac*.* Interestingly, the expression level of H3K27ac was increased with EPZ6438 treatment (Figures 2E,F).

### UNC1999 gives rise to apoptosis in uveal melanoma cells

**FIGURE 3:**
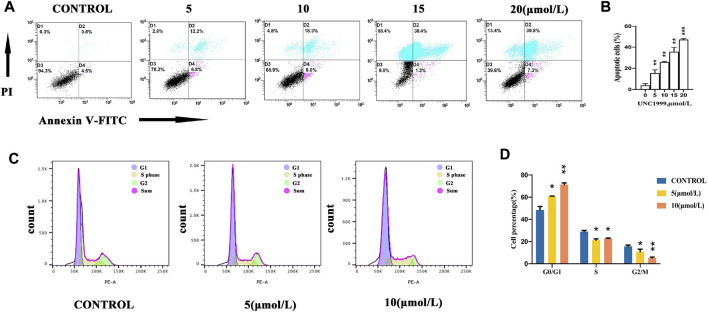
UNC1999 gives rise to apoptosis in uveal melanoma cells. **(A,B)** OMM1 cells were treated with 5, 10, 15, and 20 μM for flow cytometric analysis. **(C,D)** OMM1 cells were blocked at the S phase in both the 5 and 10 μM groups in the cell cycle. Data are expressed as mean ± SD from at least three independent experiments with triple replicates per experiment. (*p < 0.05, **p < 0.01, ***p < 0.001).

To investigate the inhibitory effect of UNC1999 in OMM1 cells, we evaluated apoptosis induced by UNC 1999. OMM1 cells were treated with four different concentrations (5, 10, 15, 20 μM) of UNC1999 for 48 h and subjected to Annexin V-FITC and PI staining for flow cytometric analysis. The results showed that the four different concentrations gave rise to 19%, 26.3%, 31.7%, and 47.1% apoptotic cells, which was statistically significant (Figures 3A,B). In the cell cycle, we found that OMM1 cells were blocked at the S phase in the 5 and 10 μM groups compared with the control group treated with UNC1999 after 48 h (Figures 3C,D).

### UNC1999 affects the cell cycle in uveal melanoma cells

**FIGURE 4:**
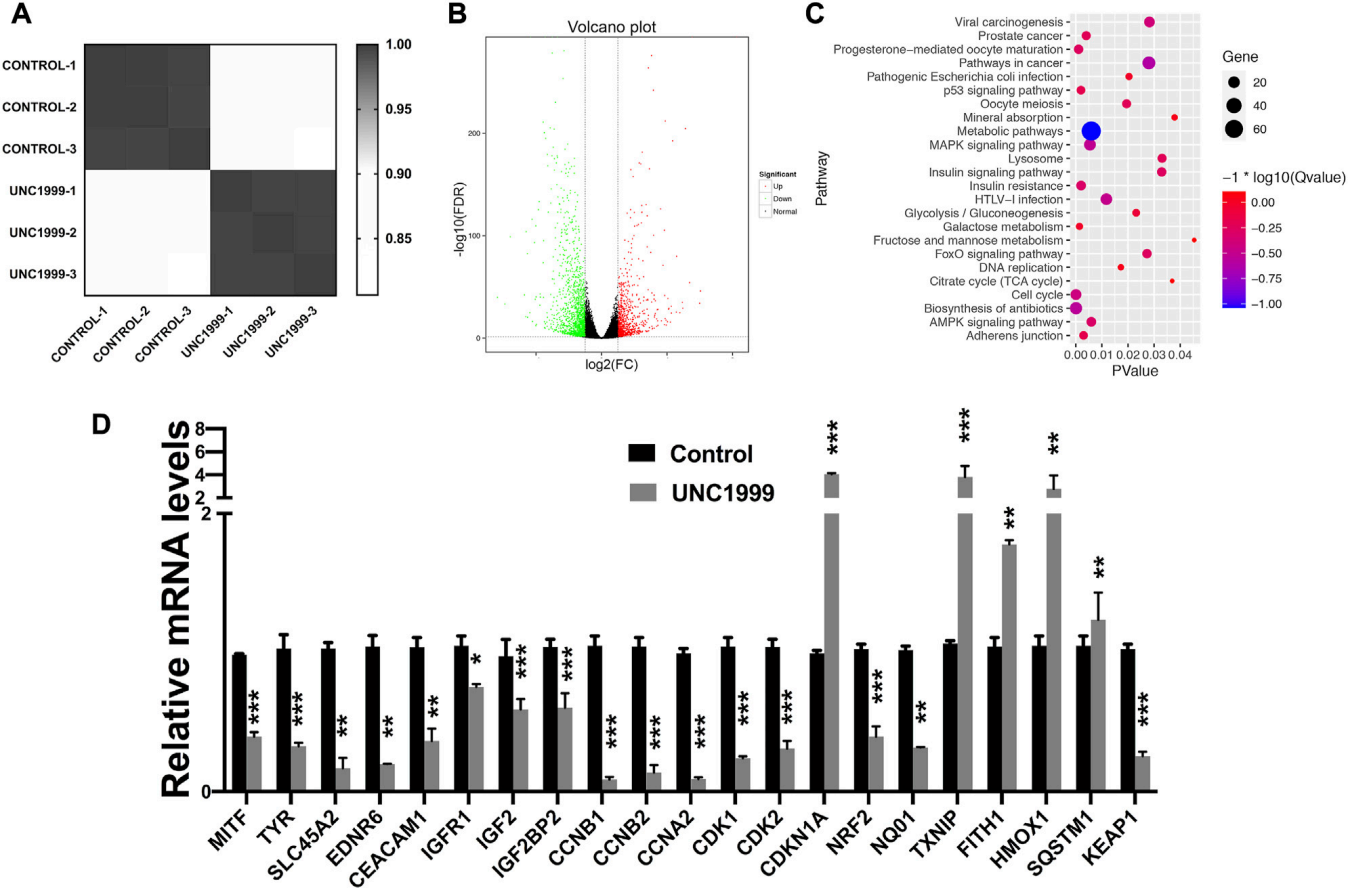
UNC1999 affects the cell cycle in OMM1 cells. **(A)** Cluster analysis of gene expression of the treatment of control and UNC 1999. **(B)** 207 upregulated genes and 449 downregulated genes in Volcano Plot. **(C)** Gene enrichment analysis using DAVID. **(D)** The relative mRNA levels of indicated DEGs and indicated treatment group was analyzed by RT-PCR. Data are expressed as mean ± SD from at least three independent experiments with triple replicates per experiment. (*p < 0.05, **p < 0.01, ***p < 0.001).

To understand how UNC1999 inhibits growth and to explore the mechanism, we performed RNA sequencing with or without UNC1999 treatment (5.0 μM) after 2 days in OMM1 cells. Six samples were evaluated, including three control samples and three samples treated with UNC1999. Average linkage hierarchical cluster analysis indicated that UNC1999 samples differed significantly from control samples, suggesting that UNC1999 treatment changed the transcriptome significantly (Figure 4A).

We analyzed differentially expressed genes (DEGs) and found 656 DEGs, including 207 upregulated genes and 449 downregulated genes (Figure 4B). All 656 DEGs were analyzed by DAVID. The results of GO analysis indicated that upregulated DEGs were primarily involved in endoplasmic reticulum stress and apoptosis. The mentioned downregulated DEGs were mainly involved in mitotic cytokinesis (Supplementary Table S4).

To better understand how DEGs worked in OMM1 cells induced by UNC1999, we analyzed KEGG pathway enrichment in DAVID. The results showed that the DEGs were particularly enriched in the cell cycle, biosynthesis of antibiotics, progesterone-mediated oocyte maturation, metabolic pathways, and p53 pathway (Figure 4C). We then focused on the cell cycle for further investigation. RT-PCR results confirmed the expression changes of *CCNB1, CCNB2, CCNA2, CDKN1A, CDK1,* and *CDK2* in the cell cycle genes, which is consistent with the RNA sequencing results (Figure 4D).

### UNC1999 induces ferroptosis in uveal melanoma

**FIGURE 5:**
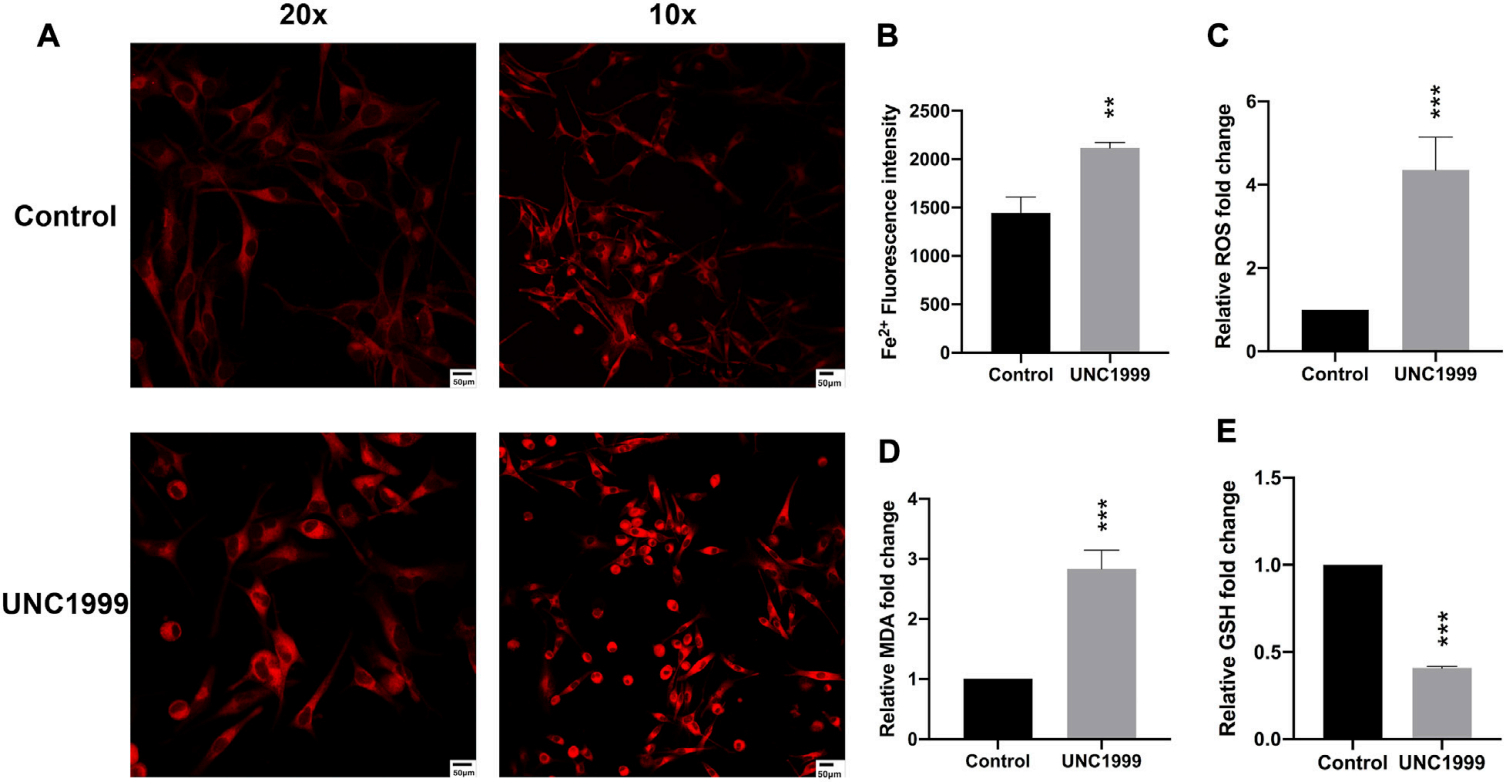
UNC1999 induced ferroptosis. **(A,B)** The intracellular iron level was increased in OMM1 cells after treatment with UNC1999. **(C–E)** ROS, MDA, and GSH content were measured in OMM1 cells after treatment with UNC 1999. Data are expressed as mean ± SD from at least three independent experiments with triple replicates per experiment. (*p < 0.05, **p < 0.01, ***p < 0.001).

**FIGURE 6:**
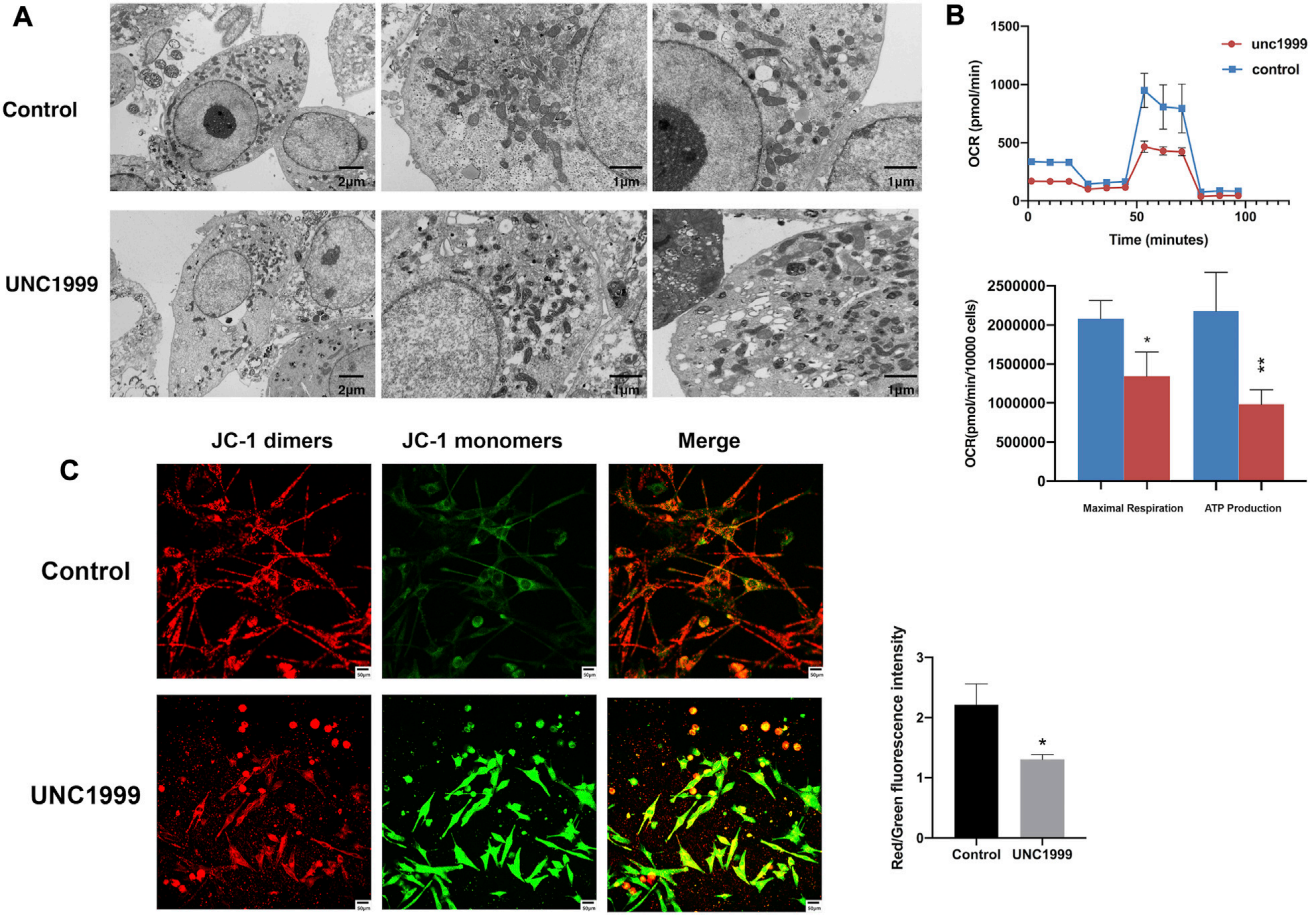
UNC1999 caused structural damage and dysfunction in mitochondria. **(A)** Transmission electron microscopy image shows the ultrastructure of OMM1 cells treated with UNC1999 for 48 h. The volume of mitochondria decreased, the outer mitochondrial membrane was disrupted, the mitochondrial ridge decreased or was absent, and membrane density in certain areas was increased in the UNC1999 group. **(B)** Oxygen consumption rate (OCR) curves and Seahorse XF analyzer measurements of mitochondrial respiration of OMM1 cells. After 48 h treatment of UNC1999, maximal respiration and ATP production were reduced. **(C)** The mitochondrial membrane potential of OMM1 cells by JC-1 assay. Data are expressed as mean ± SD from at least three independent experiments with triple replicates per experiment. (*p < 0.05, **p < 0.01, ***p < 0.001).

In the RNA sequencing and RT-qPCR results, we also noticed the different expression genes involved in ferroptosis pathways, including *HMOX1, NRF2*, *TXNIP*, *FITH1*, *SQSTM1, and KEAP1*. Further experiments verified that UNC1999 increased Fe^2+^, ROS (reactive oxygen species), MDA (malondialdehyde), and decreased GSH (glutathione) (Figures 5A–E). To verify the ferroptosis, we used transmission electron microscopy to capture the ultrastructure of OMM1 cells treated with UNC1999 for 48 h. We found the volume of mitochondria decreased, the outer mitochondrial membrane was disrupted, the mitochondrial ridge was reduced or absent, and membrane density in certain areas was increased (Figure 6A). To test the influence of metabolism on mitochondria, we measured respiration using a Seahorse XF analyzer. After 48 h treatment of UNC1999, maximal respiration and ATP production were reduced (Figure 6B). This suggested that UNC1999 can damage mitochondrial respiratory capacity. The mitochondrial potential of OMM1 cells was measured by the JC-1 Mitochondrial Membrane Potential Assay Kit. JC-1 aggregated in the mitochondrial matrix to form JC-1 dimerse of red fluorescence indicated the high mitochondrial potential of cells. On the contrary, JC-1 monomers with low mitochondrial potential were observed as green fluorescence. Red/Green fluorescence intensity was significantly decreased after treatment with UNC1999 (Figure 6C). This indicated the mitochondrial membrane potential was reduced. These findings provided direct evidence that UNC1999 can induce ferroptosis in uveal melanoma.

## Discussion

In our study, *EZH2* expression in OMM1 cells at the mRNA level was higher than in ARPE cells. In accordance with the mRNA level, EZH2 protein expression in uveal melanoma cells was higher than that in normal ocular cells, and the same result was detected for H3K27me3 expression. We investigated the expression of EZH2 and H3K27me3 in 85 UM samples. We confirmed that EZH2 (48%) and H3K27me3 (58%) were both overexpressed. Cheng and colleagues found that high EZH2 expression was associated with metastases (*p* = 0.035). In the TCGA database, the mRNA expression level of *EZH2* was correlated with the poor prognosis. But in our study, EZH2 protein expression was not associated with overall survival. This difference may result from different sample sources and our low rate of metastasis (22%) compared with theirs (35%). We also found that H3K27me3 protein expression was correlated with poor overall survival.

Previously, high H3K27me3 expression was identified as a predictor of the poor prognosis in hepatocellular carcinomas, nasopharyngeal carcinoma, urothelial carcinoma of the bladder and so on (Cai et al., 2011a; Cai et al., 2011b; Liu et al., 2013). EPZ6438 (tazemetostat), an EZH2 inhibitor, is an FDA-approved drug for epithelioid sarcoma and follicular lymphoma in 2020. There are 28 ongoing studies studying EPZ6438 alone and in combination with other medications in a phase 1/2 clinical trial (resource: https://clinicaltrials.gov/). In our research, H3K27me3 was an independent factor for the poor prognosis of UM. The overall survival of UM patients with high H3K27me3 expression was shorter than that of UM patients with low H3K27me3 expression. In addition, H3K27me3 expression was positively associated with the metastasis of UM. This phenomenon indicates that high H3K27me3 could be a biomarker predicting UM patient survival and metastasis.

Inhibitors of *EZH2* are effective in many cancers, such as lymphoma, melanoma, colorectal cancer, breast cancer, and lung cancer (Yan et al., 2017). Additionally, in Jin’s study, 92.1, Mel270 and Omm2.3, and OMM1 cell lines have a high level of expression of EZH2. And they all can be inhibited by UNC1999. Transduction by lentiviral shRNA against *EZH2* prohibited melanosphere formation and serially-replating ability in 92.1 and Mel270 cells. And depletion of *EZH2* suppressed the migration of UM cells (Jin et al., 2020). A previous study reported that *EZH1/2* dual inhibitors have more significant activity than *EZH2* selective inhibitors (Honma et al., 2017; Rizq et al., 2017). UNC1999 is an inhibitor of wild-type and mutant *EZH2* and *EZH1* (Konze et al., 2013). Compared with GSK126, GSK503, EPZ6438, and EED226, UNC1999 manifested the most potency. UNC1999 inhibited uveal melanoma cell proliferation and reduced H3K27me3 protein expression most effectively. Although *EZH2*, but not *EZH1*, is a core factor for maintaining global H3K27me3 levels, *EZH1* compensates for the function of *EZH2* in cells depleted of *EZH2* (Shen et al., 2008; Sashida and Iwama, 2017). This may be why the *EZH1/2* dual inhibitor is more active than the *EZH2* selective inhibitors GSK126, GSK503, and EPZ6438. This mechanism successfully catalyzes H3K27 methylation despite inhibition of *EZH2*.

In the present study, a high concentration of EPZ6438 had the most minor potency against cell viability. In Schoumacher’s letter, EPZ6438 (0–10 μM) treatment decreased H3K27me3 expression, showing inhibitory potency in EZH2 activity. However, it was challenging to inhibit UM cell line (92.1, MP41, MP46, MP65) growth (Schoumacher et al., 2016). Huang used 83 cancer cell lines derived from hematologic malignancies, breast cancer, liver cancer, pancreatic cancer, and lung cancer, most of which had high levels of EZH2 expression (Huang et al., 2018). After treatment with EPZ6438, they compared sensitive and insensitive cells, showing that H3K27ac levels were increased and H3K27me3 levels were decreased in insensitive cells (Huang et al., 2018). These findings indicated that upregulation of H3K27ac is associated with resistance to *EZH2* inhibition. We found that EPZ6438 could not inhibit OMM1 cell growth but could abolish the H3K27me3 protein expression. We speculated that this phenomenon was related to the increased level of H3K27ac. To further explore the reason for this effect, we found that the level of H3K27ac was upregulated after treatment with EPZ6438. This result shows that H3K27ac alteration is associated with resistance to *EZH2* inhibition in OMM1 cells. This could provide a strategy for the treatment of small molecule inhibitors of UM. We determined drug resistance according to the response to the treatment and the level of H3K27ac.

Ferroptosis, a newly programmed cell death, has been associated with breast cancer, ovarian cancer, lung cancer, renal cell carcinoma, and even involves tumorigenesis, progression, and metastasis (Xie et al., 2016; Lee et al., 2018). In the RNA sequencing and RT-qPCR results, we also found some genes associated with the ferroptosis pathway, such as *HMOX1, NRF2*, *TXNIP*, *FITH1*, *SQSTM1, and KEAP1* (Chang et al., 2018; Kim et al., 2020; Luo and Ma, 2021). Recently, two articles reported the critical role of ferroptosis in uveal melanoma by bioanalysis of public databases (Jin et al., 2021; Luo and Ma, 2021). In our study, the *HMOX1* gene expression was significantly enhanced.

Increased free iron, generation of reactive oxygen species (ROS), and accumulated lipid peroxides contribute to ferroptosis. These morphology changes are depicted by condensation of mitochondria, reduction of mitochondrial cristae, and decrease in mitochondrial size (Dixon et al., 2012). In our study, we found the inhibitor UNC1999 also can increase the level of Fe^2+^, ROS(reactive oxygen species), MDA (malondialdehyde), and decrease GSH (glutathione). At the same time, it can damage the structure and metabolism of mitochondria and contribute to ferroptosis.

In summary, we found overexpression of EZH2 and H3K27me3 in UM. This is the first study to detect the expression of H3K27me3 in UM by IHC. H3K27me3 can serve as a biomarker predicting the poor prognosis. UNC1999 can inhibit OMM1 cell growth by downregulating H3K27me3 involving the cell cycle and ferroptosis pathway. These results indicate that *EZH2* is a potential therapeutic target in UM.

## Data Availability

The datasets presented in this study can be found in online repositories. The names of the repository/repositories and accession number(s) can be found in the article/Supplementary Material.

## References

[B1] Adriana A. Rosaria G. Francesca P. Giovanna A. Gaia B. Silvano F. (2017). The biology of uveal melanoma. Cancer Metastasis Rev. 36 (1), 109–140. 10.1007/s10555-017-9663-3 28229253 PMC5385203

[B2] Bachmann I. M. Halvorsen O. J. Collett K. Stefansson I. M. Straume O. Haukaas S. A. (2006). EZH2 expression is associated with high proliferation rate and aggressive tumor subgroups in cutaneous melanoma and cancers of the endometrium, prostate, and breast. J. Clin. Oncol. 24 (2), 268–273. 10.1200/JCO.2005.01.5180 16330673

[B3] Barsotti A. M. Ryskin M. Rollins R. A. (2015). Epigenetic reprogramming in solid tumors: Therapeutic implications of EZH2 gain-of-function mutations. Epigenomics 7 (5), 687–690. 10.2217/epi.15.27 26317265

[B4] Cai G. H. Wang K. Miao Q. Peng Y. S. Chen X. Y. (2010). Expression of polycomb protein EZH2 in multi-stage tissues of gastric carcinogenesis. J. Dig. Dis. 11 (2), 88–93. 10.1111/j.1751-2980.2010.00420.x 20402834

[B5] Cai M-Y. Hou J-H. Rao H-L. Luo R-Z. Li M. Pei X-Q. (2011). High expression of H3K27me3 in human hepatocellular carcinomas correlates closely with vascular invasion and predicts worse prognosis in patients. Mol. Med. 17 (1-2), 12–20. 10.2119/molmed.2010.00103 20844838 PMC3022987

[B6] Cai M. Y. Tong Z. T. Zhu W. Wen Z. Z. Rao H. L. Kong L. L. (2011). H3K27me3 protein is a promising predictive biomarker of patients' survival and chemoradioresistance in human nasopharyngeal carcinoma. Mol. Med. 17 (11-12), 1137–1145. 10.2119/molmed.2011.00054 21738951 PMC3321814

[B7] Carvajal R. D. Schwartz G. K. Tezel T. Marr B. Francis J. H. Nathan P. D. (2017). Metastatic disease from uveal melanoma: Treatment options and future prospects. Br. J. Ophthalmol. 101 (1), 38–44. 10.1136/bjophthalmol-2016-309034 27574175 PMC5256122

[B8] Chang L. C. Chiang S. K. Chen S. E. Yu Y. L. Chou R. H. Chang W. C. (2018). Heme oxygenase-1 mediates BAY 11-7085 induced ferroptosis. Cancer Lett. 416, 124–137. 10.1016/j.canlet.2017.12.025 29274359

[B9] Cheng Y. Li Y. Huang X. Wei W. Qu Y. (2017). Expression of EZH2 in uveal melanomas patients and associations with prognosis. Oncotarget 8 (44), 76423–76431. 10.18632/oncotarget.19462 29100322 PMC5652716

[B10] Colón-Caraballo M. Monteiro J. B. Flores I. (2015). H3K27me3 is an epigenetic mark of relevance in endometriosis. Reprod. Sci. 22 (9), 1134–1142. 10.1177/1933719115578924 25820690 PMC5590701

[B11] Divakaruni A. S. Paradyse A. Ferrick D. A. Murphy A. N. Jastroch M. (2014). Analysis and interpretation of microplate-based oxygen consumption and pH data. Methods Enzymol. 547, 309–354. 10.1016/B978-0-12-801415-8.00016-3 25416364

[B12] Dixon S. J. Lemberg K. M. Lamprecht M. R. Skouta R. Zaitsev E. M. Gleason C. E. (2012). Ferroptosis: An iron-dependent form of nonapoptotic cell death. Cell 149 (5), 1060–1072. 10.1016/j.cell.2012.03.042 22632970 PMC3367386

[B13] Ernst T. Chase A. J. Score J. Hidalgo-Curtis C. E. Bryant C. Jones A. V. (2010). Inactivating mutations of the histone methyltransferase gene EZH2 in myeloid disorders. Nat. Genet. 42 (8), 722–726. 10.1038/ng.621 20601953

[B14] Hakozaki M. Hojo H. Tajino T. Yamada H. Kikuchi S. Konno S. (2013). Poorly differentiated synovial sarcoma showing homer-wright rosette structures: A potential diagnostic pitfall. APMIS acta pathologica, Microbiol. Immunol. Scand. 121 (4), 359–361. 10.1111/j.1600-0463.2012.02964.x 23030377

[B15] Honma D. Kanno O. Watanabe J. Kinoshita J. Hirasawa M. Nosaka E. (2017). Novel orally bioavailable EZH1/2 dual inhibitors with greater antitumor efficacy than an EZH2 selective inhibitor. Cancer Sci. 108 (10), 2069–2078. 10.1111/cas.13326 28741798 PMC5623739

[B16] Huang X. Yan J. Zhang M. Wang Y. Chen Y. Fu X. (2018). Targeting epigenetic crosstalk as a therapeutic strategy for EZH2-aberrant solid tumors. Cell 175 (1), 186–199. 10.1016/j.cell.2018.08.058 30220457

[B17] Jin B. Zhang P. Zou H. Ye H. Wang Y. Zhang J. (2020). Verification of EZH2 as a druggable target in metastatic uveal melanoma. Mol. Cancer 19 (1), 52. 10.1186/s12943-020-01173-x 32127003 PMC7055080

[B18] Jin Y. Wang Z. He D. Zhu Y. Gong L. Xiao M. (2021). Analysis of ferroptosis-mediated modification patterns and tumor immune microenvironment characterization in uveal melanoma. Front. Cell Dev. Biol. 9, 685120. 10.3389/fcell.2021.685120 34386492 PMC8353259

[B19] Jung H. R. Pasini D. Helin K. Jensen O. N. (2010). Quantitative mass spectrometry of histones H3.2 and H3.3 in Suz12-deficient mouse embryonic stem cells reveals distinct, dynamic post-translational modifications at Lys-27 and Lys-36. Mol. Cell. Proteomics 9 (5), 838–850. 10.1074/mcp.M900489-MCP200 20150217 PMC2871418

[B20] Kahn T. G. Dorafshan E. Schultheis D. Zare A. Stenberg P. Reim I. (2016). Interdependence of PRC1 and PRC2 for recruitment to polycomb response elements. Nucleic Acids Res. 44 (21), 10132–10149. 10.1093/nar/gkw701 27557709 PMC5137424

[B21] Katz L. M. Hielscher T. Liechty B. Silverman J. Zagzag D. Sen R. (2018). Loss of histone H3K27me3 identifies a subset of meningiomas with increased risk of recurrence. Acta Neuropathol. 135 (6), 955–963. 10.1007/s00401-018-1844-9 29627952

[B22] Kim J. Koo B. K. Knoblich J. A. (2020). Human organoids: Model systems for human biology and medicine. Nat. Rev. Mol. Cell Biol. 21 (10), 571–584. 10.1038/s41580-020-0259-3 32636524 PMC7339799

[B23] Konze K. D. Ma A. Li F. Barsyte-Lovejoy D. Parton T. Macnevin C. J. (2013). An orally bioavailable chemical probe of the Lysine Methyltransferases EZH2 and EZH1. ACS Chem. Biol. 8 (6), 1324–1334. 10.1021/cb400133j 23614352 PMC3773059

[B24] Lee S. Y. Ju M. K. Jeon H. M. Jeong E. K. Lee Y. J. Kim C. H. (2018). Regulation of tumor progression by programmed necrosis. Oxid. Med. Cell. Longev. 2018, 3537471. 10.1155/2018/3537471 29636841 PMC5831895

[B25] Li Z. Zhao S. Zhu S. Fan Y. (2021). MicroRNA-153-5p promotes the proliferation and metastasis of renal cell carcinoma via direct targeting of AGO1. Cell Death Dis. 12 (1), 33. 10.1038/s41419-020-03306-y 33414440 PMC7791042

[B26] Liu F. Gu L. Cao Y. Fan X. Zhang F. Sang M. (2016). Aberrant overexpression of EZH2 and H3K27me3 serves as poor prognostic biomarker for esophageal squamous cell carcinoma patients. Biomarkers 21 (1), 80–90. 10.3109/1354750X.2015.1118537 26631178

[B27] Liu J. Li Y. Liao Y. Mai S. Zhang Z. Liu Z. (2013). High expression of H3K27me3 is an independent predictor of worse outcome in patients with urothelial carcinoma of bladder treated with radical cystectomy. Biomed. Res. Int. 2013, 390482. 10.1155/2013/390482 24093096 PMC3777191

[B28] Liu J. Liang L. Huang S. Nong L. Li D. Zhang B. (2019). Aberrant differential expression of EZH2 and H3K27me3 in extranodal NK/T-cell lymphoma, nasal type, is associated with disease progression and prognosis. Hum. Pathol. 83, 166–176. 10.1016/j.humpath.2018.08.025 30218753

[B29] Liu X. Liu X. (2022). PRC2, chromatin regulation, and human disease: Insights from molecular structure and function. Front. Oncol. 12, 894585. 10.3389/fonc.2022.894585 35800061 PMC9255955

[B30] Luo H. Ma C. (2021). A novel ferroptosis-associated gene signature to predict prognosis in patients with uveal melanoma. Diagn. (Basel, Switz. 11 (2), 219. 10.3390/diagnostics11020219 PMC7913108 33540700

[B31] McCabe M. T. Graves A. P. Ganji G. Diaz E. Halsey W. S. Jiang Y. (2012). Mutation of A677 in histone methyltransferase EZH2 in human B-cell lymphoma promotes hypertrimethylation of histone H3 on lysine 27 (H3K27). Proc. Natl. Acad. Sci. U. S. A. 109 (8), 2989–2994. 10.1073/pnas.1116418109 22323599 PMC3287005

[B32] Miller K. Hall R. C. Brenn T. (2012). Spitz nevus with Homer-Wright rosette-like structures. Am. J. Dermatopathol. 34 (4), 457–459. 10.1097/DAD.0b013e31823b9caf 22356919

[B33] Ngollo M. Lebert A. Daures M. Judes G. Rifai K. Dubois L. (2017). Global analysis of H3K27me3 as an epigenetic marker in prostate cancer progression. BMC cancer 17, 261. 10.1186/s12885-017-3256-y 28403887 PMC5388998

[B34] Nikoloski G. Langemeijer S. M. Kuiper R. P. Knops R. Massop M. Tönnissen E. R. (2010). Somatic mutations of the histone methyltransferase gene EZH2 in myelodysplastic syndromes. Nat. Genet. 42 (8), 665–667. 10.1038/ng.620 20601954

[B35] Ntziachristos P. Tsirigos A. Van Vlierberghe P. Nedjic J. Trimarchi T. Flaherty M. S. (2012). Genetic inactivation of the polycomb repressive complex 2 in T cell acute lymphoblastic leukemia. Nat. Med. 18 (2), 298–301. 10.1038/nm.2651 22237151 PMC3274628

[B36] Peters A. H. Kubicek S. Mechtler K. O'Sullivan R. J. Derijck A. A. Perez-Burgos L. (2003). Partitioning and plasticity of repressive histone methylation states in mammalian chromatin. Mol. Cell 12 (6), 1577–1589. 10.1016/s1097-2765(03)00477-5 14690609

[B37] Rizq O. Mimura N. Oshima M. Saraya A. Koide S. Kato Y. (2017). Dual inhibition of EZH2 and EZH1 sensitizes PRC2-dependent tumors to proteasome inhibition. Clin. Cancer Res. 23 (16), 4817–4830. 10.1158/1078-0432.CCR-16-2735 28490465 PMC5562278

[B38] Sashida G. Iwama A. (2017). Multifaceted role of the polycomb-group gene EZH2 in hematological malignancies. Int. J. Hematol. 105 (1), 23–30. 10.1007/s12185-016-2124-x 27830540

[B39] Schaefer I. M. Fletcher C. D. Hornick J. L. (2016). Loss of H3K27 trimethylation distinguishes malignant peripheral nerve sheath tumors from histologic mimics. Mod. Pathol. 29 (1), 4–13. 10.1038/modpathol.2015.134 26585554

[B40] Schoumacher M. Le Corre S. Houy A. Mulugeta E. Stern M-H. Roman-Roman S. (2016). Uveal melanoma cells are resistant to EZH2 inhibition regardless of BAP1 status. Nat. Med. 22 (6), 577–578. 10.1038/nm.4098 27270772

[B41] Shen X. Liu Y. Hsu Y. J. Fujiwara Y. Kim J. Mao X. (2008). EZH1 mediates methylation on histone H3 lysine 27 and complements EZH2 in maintaining stem cell identity and executing pluripotency. Mol. Cell 32 (4), 491–502. 10.1016/j.molcel.2008.10.016 19026780 PMC2630502

[B42] Wei Y. Xia W. Zhang Z. Liu J. Wang H. Adsay N. V. (2008). Loss of trimethylation at lysine 27 of histone H3 is a predictor of poor outcome in breast, ovarian, and pancreatic cancers. Mol. Carcinog. 47 (9), 701–706. 10.1002/mc.20413 18176935 PMC2580832

[B43] Xie Y. Hou W. Song X. Yu Y. Huang J. Sun X. (2016). Ferroptosis: Process and function. Cell Death Differ. 23, 369–379. 10.1038/cdd.2015.158 26794443 PMC5072448

[B44] Yan K. S. Lin C. Y. Liao T. W. Peng C. M. Lee S. C. Liu Y. J. (2017). EZH2 in cancer progression and potential application in cancer therapy: A friend or foe? Int. J. Mol. Sci. 18 (6), E1172. 10.3390/ijms18061172 28561778 PMC5485996

[B45] Yonekawa Y. Kim I. K. (2012). Epidemiology and management of uveal melanoma. Hematol. Oncol. Clin. North Am. 26 (6), 1169–1184. 10.1016/j.hoc.2012.08.004 23116575

