# Supplementary figures and images for: EZH2-mediated H3K27me3 is a predictive biomarker and therapeutic target in uveal melanoma
Source: Front Genet. 2022 Oct 6;13:1013475. doi: 10.3389/fgene.2022.1013475 (PMC9582331; doi:10.3389/fgene.2022.1013475)

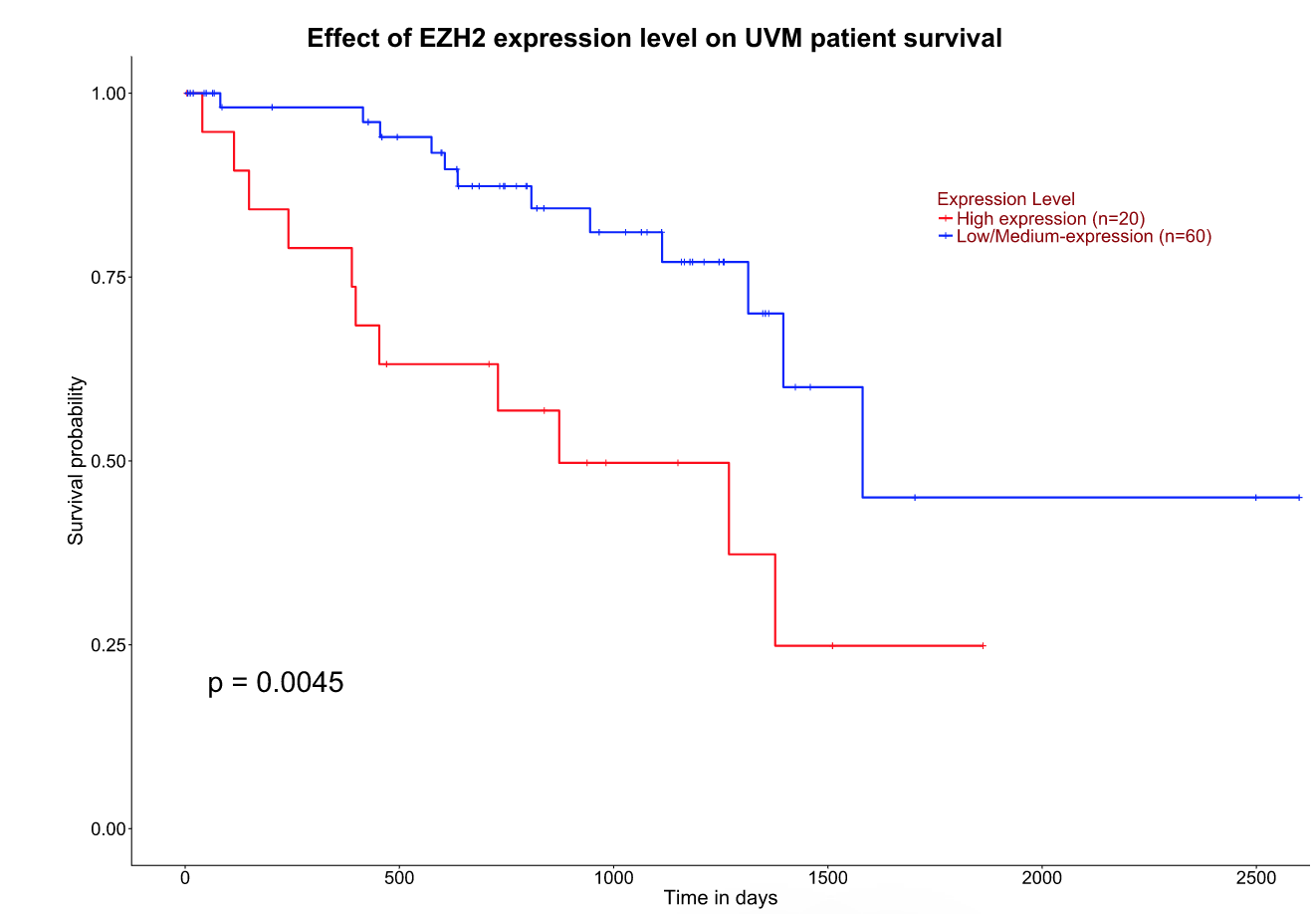

Supplement: Supplementary file 5 [file Image1.PNG]
