# Supplementary material for: EZH2-mediated H3K27me3 is a predictive biomarker and therapeutic target in uveal melanoma
Source: Front Genet. 2022 Oct 6;13:1013475. doi: 10.3389/fgene.2022.1013475 (PMC9582331; doi:10.3389/fgene.2022.1013475)
Supplement: Supplementary file 6 [file DataSheet1.docx]

The Raw Data can be seen at: https://www.jianguoyun.com/p/DQ43q_kQyMboChjRg9EEIAA.
